# Digital health—high tech or high touch?

**DOI:** 10.1007/s10354-022-00991-6

**Published:** 2023-01-05

**Authors:** Clemens Gangl, Konstantin Krychtiuk

**Affiliations:** Department of Internal Medicine II, Division of Cardiology, Medical University of Vienna, Währinger Gürtel 18–20, 1090 Vienna, Austria

**Keywords:** Artificial intelligence, Machine learning, Deep neural networks, Digital therapeutics, Telemedicine, Künstliche Intelligenz, Maschinelles Lernen, Tiefe neuronale Netze, Digitale Therapeutika, Telemedizin

## Abstract

Digital transformation in medicine refers to the implementation of information technology-driven developments in the healthcare system and their impact on the way we teach, share, and practice medicine. We would like to provide an overview of current developments and opportunities but also of the risks of digital transformation in medicine. Therefore, we examine the possibilities wearables and digital biomarkers provide for early detection and monitoring of diseases and discuss the potential of artificial intelligence applications in medicine. Furthermore, we outline new opportunities offered by telemedicine applications and digital therapeutics, discuss the aspects of social media in healthcare, and provide an outlook on “Health 4.0.”

## Abstract (de)

Digitale Transformation der Medizin bezieht sich auf den Einzug von Entwicklungen der Informationstechnologie in das Gesundheitswesen und deren Auswirkungen auf die Art und Weise, wie Medizin gelehrt und praktiziert wird. Wir wollen einen Überblick über aktuelle Entwicklungen, Chancen, aber auch Risiken der digitalen Transformation in der Medizin geben. Dafür beleuchten wir die Möglichkeiten, die Wearables und digitale Biomarker in der Früherkennung und Überwachung von Krankheiten bieten, und diskutieren das Potenzial von Anwendungen künstlicher Intelligenz in der Medizin. Darüber hinaus werden telemedizinische Anwendungen und digitale Therapeutika dargestellt, Aspekte von Social Media im Gesundheitswesen beschrieben, und es wird ein Ausblick auf „Gesundheit 4.0“ gegeben.

## Introduction

Our healthcare system faces major challenges, necessary changes, but most importantly, unique opportunities. The Coronavirus disease 2019 (COVID-19) pandemic has provided an unprecedented boost for many transformations and disrupted several of our traditional approaches. Within days, whole clinics were forced to switch to remote care and, in order to relieve the burden on outpatient facilities, expanded their remote monitoring programs [1]. In areas struck with large caseloads of COVID-19, physicians and other healthcare professionals quickly connected on social media and other platforms to share cases and clinical experience as well as diagnostic and treatment algorithms and to collaborate in rapidly setting up clinical trial protocols [2]. Besides increases in efficiency and the facilitations in daily medical care, digital transformation and the collection of “big data” now allow large-scale scientific analyses, which in the past usually required large investments and expenditures. Widespread use of health apps and so-called wearables creates an immense quantity of digital biomarkers each second and, if integratively analyzed, may allow early detection of subclinical changes in health and prediction of later diseases [3].

Remote monitoring of implanted devices such as pacemakers and defibrillators dramatically reduces unnecessary outpatient visits on the one hand, and on the other hand, may signal potentially life-threatening aberrations at an early stage and trigger lifesaving medical interventions [4]. Artificial intelligence as well as machine learning and its applications exhibit a large potential in automatic imaging detection of diseases and understanding of complex connections. Other potential applications in the near future include the analysis of large datasets and application of personalized medical treatments based on digital and biochemical biomarkers also within the field of pharmaceutics [5, 6].

In light of the pandemic and stay-at-home orders, digital teaching has experienced a lot of push, and within a few weeks of the pandemic situation, online meetings and educational activities became the norm. Social media use within the scientific and medical community boomed, and now provides unprecedented possibilities for education, communication, and collaboration. But can that replace personal communication? Can medical education be delivered within 280 characters of short “tweets”? What are the risks of big health data in the hands of private companies? Who will be reliable for the mistakes of the digital radiologist? Herein, we would like to provide an overview of current developments and chances but also of the risks of digital transformation in medicine.

We believe that if used purposefully, digital technologies have immense potential for supporting physicians and other healthcare personnel with some of the time-consuming repetitive tasks that they are tasked and overwhelmed with, in helping the scientific field to understand complex relationships in big data, and, ultimately, in allowing physicians to focus on their main task: working with their patients.

## Case presentation

*To make several of the discussed areas of digital medicine more vivid, we will accompany a fictious patient with heart failure on his journey through a partially digitalized medical system in 2022. Heart failure is a chronic disease with well-studied and efficient medications and device-based treatments available* [7]. *A major focus in treating this disease is to prevent episodes of decompensation and complications, which are associated with cost- and treatment-intensive hospitalizations and worsening in the baseline status after recompensation. Our 55-year-old patient with chronic heart failure is on guideline-recommended adequately dosed medication and remained stable over several years. Over the past 2 weeks, however, he has felt a sudden worsening of performance and an increasing shortness of breath. On his 56th birthday, his nephew gave him a smartwatch to better follow up on his leisure activities. Shortly after putting on his new gift, he received an alarm for atrial fibrillation. He presented to the cardiology outpatient clinic where the rhythm disorder was confirmed, and he was admitted to the cardiology ward due to decompensation.*

## Wearables and digital biomarkers

Wearable health sensors, often integrated into smartwatches, wristbands, rings, or smart patches, facilitate close monitoring of specific body parameters. These digital biomarkers can be gathered continuously during different times of the day and in various circumstances, and may therefore create a more representative patient image compared to occasional measurements conducted during single office visits or by conventional self-monitoring strategies [8]. This potentially allows an early detection of subclinical changes in health and prediction of later diseases.

Photoplethysmography (PPG)-based detection of the pulse and thereof derived heart rate measurements are among the most common applications of wearable health sensors. A photodiode sensor integrated into the wearable device emits light into the capillary bed and detects the pulse-related change in blood volume by measuring wavelength alterations of the reflected light. Since this technology is also used in pulse oximetry, it is clinically validated for heart rate measurements and is already integrated into many devices on the wearable market [9]. However, a study has demonstrated that the signal quality might be significantly reduced in patients with darker skin tones or obesity [10]. In addition, arrhythmia diagnostics can also be performed based on a PPG-recorded pulse signal, although this approach has methodological limitations due to the absence of electrocardiographic information. Some study groups and manufacturers try to compensate for this with machine learning-based signal analysis approaches [11]. However, the advantage of PPG-based arrhythmia diagnostic methods is that they can be applied in the background without active patient interaction and may therefore detect asymptomatic episodes of atrial fibrillation.

Some devices even allow the acquisition of electrocardiographic (ECG) signals. For example, a single-channel ECG vector can be obtained by using a smartwatch’s metal bottom part touching the wrist as one electrode and connecting it to a second electrode integrated into a hardware button on the device surface. This requires active user interaction by placing one finger of the opposite hand on it. However, the thereby gained electrocardiographic signal facilitates a methodically correct detection of atrial fibrillation. This method and its implementations were evaluated in large feasibility trials conducted by several device manufacturers; some have already received approval as medical devices for the detection of atrial fibrillation by the FDA [12–14].

Today, PPG-based blood pressure measurements do not provide an equivalent alternative to conventional cuff-based devices regarding their accuracy and are currently more likely to be classified as consumer products. Further development is needed to achieve the accuracy required for reliable medical blood pressure monitoring [15]. However, cuff-based devices are available that can store measured data in cloud-based services for further analysis after being connected to a smartphone. Other exemplary applications are continuous glucose measurement using smart patches [16], sleep apnea tracking using PPG sensors [17], and built-in accelerometers for fall detection or to monitor the course of Parkinson’s disease [18]. Data access and protection are key issues, since health data gathered via wearables are often stored in proprietary cloud services and access to these by treating physicians is currently mostly unregulated. Appropriate legal frameworks must be in place to protect patients from data misuse.

*As the patient fulfilled the criteria for implantation of a three-chamber pacemaker and defibrillator, implantation was performed the day before discharge* [7]. *He was asked to participate in a remote-monitoring option for his implanted pacemaker for early detection of decompensation and rhythm disorders as well as in a remote, app-based outpatient program. Within the program, the patient is asked to record his weight, blood pressure, heart rate, and signs and symptoms of heart failure. After a few weeks of clinical stability, an increase in body weight was recorded and forwarded to a specialized heart failure nurse who then adapted the medication with the patient in a remote visit.*

## Telemedicine and digital therapeutics

Telemedical applications have experienced an enormous expansion, not least due to the COVID-19 pandemic [1]. Because of today’s broad availability and wide acceptance of teleconferencing technologies, certain parts of the medical workflow, such as anamnesis, discussion of results, or periodic follow-ups, can alternatively take place in the form of teleconsultations instead of physical in-person meetings. This not only enables low-threshold access to specialized health resources from rural areas but also allows consideration of the special needs of patients with impaired mobility. In a 2021 member survey conducted by the American Medical Association, 85% of the responding physicians indicated the use of telehealth applications, with more than 80% stating “facilitating patients better access to care” as their main motivation [19].

Remote monitoring of implanted devices such as pacemakers and implantable cardioverter-defibrillators (ICD) enables early detection of even subclinical events and, thus, allows timely, potentially lifesaving interventions. Furthermore, periodic routine device checkups that have previously taken place in an in-person outpatient setting can be performed remotely. Therefore, devices connect automatically to small home-monitoring base stations, for example, located in patients’ bedrooms, to transmit recorded anomalies to a control server via a secured cellular network connection. This allows timely assessment of potential medical events or device malfunctions by healthcare professionals [4]. Real-time monitoring and thus integration into emergency medical services is currently not possible that way. In the future, AI-supported methods of signal analysis may help to prioritize the emerging number of reported events more efficiently.

In addition to securing data transmission and data storage, there are concerns about security issues affecting the direct device safety. Since the data transmission is one-way only (read-only for remote monitoring), the security threat is theoretically limited to the interception of transmitted data by unauthorized persons. A potential harmful reconfiguration of device parameters by compromising the remote-monitoring connection seems therefore technically not feasible.

*Digital therapeutics* (DTx) are digital healthcare solutions that apply software-driven interventions—sometimes combined with hardware sensors—to prevent, manage, or treat diseases [20]. Although typically accessed using smartphones or tablet computers, these products differ from consumer-targeted health applications like fitness trackers, calory counters, step counters, or nutrition diaries, as they aim to perform evidence-based therapeutic interventions on patients and therefore need to be approved by regulatory bodies after proving clinical efficacy and safety. These solutions may be implemented as stand-alone approaches or combined with pharmacological therapies to enhance their effects, e.g., optimize their intake or dosage, for example, combined with a hardware push sensor on inhalers in treatment of chronic obstructive pulmonary disease. DTx cover an expanding range of medical applications, including managing and accompanying treatment of chronic diseases like cancer, diabetes, multiple sclerosis, or Parkinson’s disease [21]. But also the area of mental health offers a wide range of applications for disorders such as depression or anxiety. In many of these applications, there is a focus on accurately recording the course of symptoms and providing treatment recommendations based on that information. In addition to direct therapeutic effects, DTx can promote the patient’s disease awareness and thus indirectly increase patient autonomy. As of today, the German Federal Institute for Drugs and Medical Devices lists 35—partly temporarily—approved DTx applications for which reimbursements are offered in Germany [22]*. *Because data collected by DTx are often stored in vendor-owned systems, access to these data by attending physicians must be an integral part of the solution. This can be achieved by alternatively storing data into public electronic health record systems or providing data access on the base of public data exchange standards (e.g., HL7) in the future.

*After a few years of clinical stability, our patient experienced increasing dyspnea symptoms. As remote monitoring of his CRT system revealed no recorded episodes of cardiac arrhythmias, the patient visited an emergency room after consulting with his treating cardiologist. Based on the patient’s symptoms and medical history, a coronary CT scan was performed to rule out coronary ischemia. A machine learning-based diagnostic model was able to rule out significant coronary stenosis. However, his medication was adjusted for treatment of bilateral pleural effusions and a clinical follow-up was scheduled.*

## Artificial intelligence and machine learning in medicine

Machine learning, presumably the most prominent subfield of artificial intelligence these days, refers to strategies for solving defined tasks in which computers are not explicitly programmed with rule-based algorithms, but are taught on an abstract level to acquire a solution from existing data. These strategies aim to develop a mathematical function that can predict an output value based on a number of input values, for example, to classify a patient’s condition into “critically ill” or “recovering,” or to predict a numerical value such as the duration of treatment based on several acquired values. Due to the large number of input values (several thousand to even millions) that arise in some applications such as image processing, the derived functions are often rather complex. An essential factor in building machine learning models is whether training data with corresponding output values are available during the training phase. As this facilitates an iterative refinement process based on a feedback mechanism while building the model, such strategies are known as *supervised learning* strategies. If no annotated training data are available, *unsupervised learning* strategies can be applied to classify datasets based on identified similarities or detected anomalies.

Especially advances in the field of *artificial neural networks* (ANNs) and their sub-form *convolutional neural networks* (CNNs) have contributed to the rapid spread of machine learning-based applications in healthcare. ANNs are complex mathematical functions that model processes in the human cerebral cortex in an abstract way. Each ANN is composed of one input and one output layer which encompass several so-called hidden layers of artificial neurons between them. By passing a set of input values through these successive layers of neurons, features can be extracted in an incremental way. For example, an ANN trained on certain images could learn to identify edges in its first layers, shapes based on these edges in further layers, and specific objects based on these shapes in its final layers. The functionality and performance of the network result from the pattern in which the neurons are interconnected. This pattern develops during the training process by tweaking the weights and biases that are associated with these connections. Depending on the complexity of the task, a considerable amount of training data may therefore be required. Network architectures with more than two hidden layers are referred to as *deep neural networks* (DNN).

Since Esteva et al. demonstrated in a remarkable 2017 publication that a CNN trained on nearly 130,000 images performed at least equally well in classifying lesions suspicious for skin cancer compared to board-certified dermatologists, the field of machine learning-based applications in medicine experienced rapid further development, with more than 85,000 listed publications in PubMed as of today [23].

Because these approaches perform particularly well in pattern-recognition tasks, various studies have been published especially in the fields of radiology and signal analysis. Examples include publications about (COVID-19) pneumonia detection or segmentation of pulmonary embolism in thoracic CT images [24, 25]. Interesting applications also arise in signal analysis. Apart from studies on the detection of atrial fibrillation based on ECG and PPG signals [11], machine learning-based approaches can also emphasize disease markers that may have not had significance in clinical practice yet. For example, a study by Attia et al. demonstrated that individuals with paroxysmal atrial fibrillation may also be identified even during phases of sinus rhythm by analyzing standard 12-lead ECGs. The pathophysiological background is presumably based on specific patterns in the P‑wave section and alterations of the PQ interval [26].

However, this study also highlights an important limitation of neural network classifier models—the lack of explainability. Since the processes that arise during the building of ANNs reach an enormous level of complexity, the traceability of their decisions is currently virtually impossible. Although a lot of scientific effort goes into researching “explainable AI,” ANNs have a reputation for resembling a black box. Aside from legal product-liability issues, this also raises concerns about trustworthiness, which is an important factor for its clinical acceptance. Both science and industry are required to address these issues. Other important concerns are potential gender and ethnic biases of ANNs, which may occur when training datasets underrepresent certain patient populations. The impact of this topic has been demonstrated by several studies to date. Thoughtful selection of training data and awareness of this issue in clinical application is of absolute necessity [27–30].

In addition to their application in diagnostics, machine learning-based approaches can help predict the risk of disease development or certain courses for individual patients (“predictive AI”). This may help to reduce the onset of certain diseases through individually tailored screening and preventive measures and to deploy resources in a more targeted manner. Used wisely, machine learning-based applications will empower physicians by taking over the burden of repetitive low-level tasks to provide time for more demanding tasks and, not least, personal patient contact.

## Social media in medicine

Within the past decade, social media has become ubiquitous within our society with more than 3.5 billion users worldwide using a variety of platforms and a clear prediction for further growth [31]. Users of social media use its platforms for a large variety of reasons such as a news source, a social tool, a place to conduct business, and as a source of health information. As basically all aspects of life are, to a certain extent, represented in social media, so are medical information, discussion, and education as well as medical providers themselves. A search for “social media AND medicine” on PubMed.gov revealed 6500 results up until 2018, since then the number of results has more than doubled (16,183 as of September 30, 2022)—evidence for a strong uptake of social media within the medical community. Within the past years, several scandals have shaken the social media world and highlighted important vulnerabilities [32], including potential efforts to influence elections with the use of so-called bots [33, 34]. A seminal analysis published in *Science* assessed the spread of true and false news online [35]. The authors demonstrated that false information spread faster, farther, deeper, and more broadly as compared to true information. Importantly, bots spread true and false information at the same rate, suggesting that it is actually humans who more likely spread falsehoods on social media. This begs the question, is social media really the right place for scientific discussion and medical education?

### From congress coverage …

One area of interest for social media in medicine is congress coverage: up until recently, medical scientific meetings were in-person meetings only and if one could not attend, reading summaries and important publications was the only way to catch up. Therefore, congress coverage was probably one of the first areas where medical social media, in particular the microblogging service Twitter, flourished. An analysis of the European Society of Cardiology congress of 2018 showed that more than 55,000 “tweets”, short messages, were created by more than 12,000 participants within and outside of the meeting [36]. A detailed analysis suggested that more than 80% of such tweets were of educational nature. What are some of the advantages of social media use at medical conferences? It allows rapid, timely, and critical discussion of novel research findings and presented treatment guidelines. In particular, it allows the “regular” provider or scientist to engage in a discussion with peers and leaders of the field in real time without barriers, as opposed to a predefined discussion of opinion leaders only, where regular attendees can only listen and not engage. In addition, it gives early career researchers a stage for “promotion” of their original research to be presented at the respective meeting and for discussion with opinion leaders and peers. It can thus provide leverage for scientific discussion and collaboration and connect like-minded people with similar research interests without commonly encountered barriers. One example of fruitful collaboration facilitated by social media was a project that was initiated by a young fellow who reached out to a senior expert via social media, stayed at the senior’s institution, and analyzed collaborative patient data from other experts whom the senior expert only knew via social media and published in the *American Journal of Cardiology *[37, 38].

### ... to scientific debate

In academia, scholarly peer review is the method of choice for assessing the suitability of a research paper to be accepted for publication in a scientific journal. It is a time-consuming and (usually) anonymous process, leaving other experts or doctors and researchers in training out of the process, who may, in order to discuss or (legitimately) criticize the paper after publication, send a letter to the journal which may or may not be accepted, which then, in turn, the authors may answer. Such a letter-based process stems from different times and is antiquated. Today, without having any official rules or consequences, papers undergo a form of second review by the medical and scientific community after publication by the means of social media. Two examples from the cardiovascular world deserve mention: after presentation of the ORBITA trial at the Transcatheter Cardiovascular Therapeutics meeting in 2017, which suggested that stenting of a coronary vessel with severe stenosis does not improve symptoms of angina, #CardioTwitter (a hashtag commonly used by cardiovascular professionals on social media), literally exploded [39, 40]. In the first 9 days, over 1700 English-language tweets by more than 600 users were posted, a number that by far exceeds typical citations, even for a practice-changing clinical trial. The slow uptake of the ORBITA results by new guidelines was later heavily debated, another proof of the importance of democratization made possible by social media. Another big controversy arose over the way to treat stenosis of the left main artery, the largest coronary artery supplying a large part of the heart. A large study compared coronary stenting with bypass surgery in patients with stenosis of the left main artery. Several aspects of the trial, including choice of statistical methods and endpoints, caused a huge online and offline debate involving the key opinion leaders [41]. For fellows in training, it was an incredible opportunity to learn details of trial methodology and statistical analyses from the “live” discussions arising on social media. While a tweet on Twitter can only contain 280 characters, several tweets can be put in a row, creating a so-called tweetorial. Two of such regarding the controversy mentioned before should be highlighted and are fantastic examples of “5 min online continuing education” for busy clinicians [42, 43].

### Continuing medical education

The COVID-19 pandemic has disrupted the way we teach and learn, from classical education in schools and universities to medical education and training. In times of social distancing, people gathered even more on social media platforms. With the number of physicians and physicians in training active on social media rising, social media may be a powerful tool with the potential of transforming continuing medical education (CME) [44, 45]. Increasing time pressure on physicians during their active time in the hospital makes asynchronous learning by means of social media an attractive way of learning, allowing access to educational content at any time, place, and pace [45]. Social media allows a more democratic form of education, with fewer barriers to engaging in medical and scientific discussions. This new and interactive way of learning is different and potentially more stimulating as compared to passive absorption of material associated with traditional education [44]. Hashtags and social media representation of major medical societies and journals make it easy to follow up on the newest developments [46].

What other types of learning can be found on Twitter? Twitter journal clubs, some with official CME credits, allow global discussion of novel scientific findings, reaching audiences several fold larger than conventional, localized journal clubs [47]. The option of tweetorials, providing high-quality education in an accessible format in small pieces, is yet another form of quick, “on the go” continuous education. Two great examples of tweetorials, one dedicated to an imaging form, the other focusing on the clinician interested in starting with twitter are provided [48, 49].

The unprecedented challenges that arose with the global outbreak of COVID-19 channeled a lot of discussion, sharing of experiences and knowledge, and collaborative approaches to setting up trial protocols onto social media. Webinars in addition to publication of expedited research and reviews critically relied on social media sharing.

### Discussing novel techniques

During the past decade, a new approach of performing a coronary angiogram was developed using the radial artery instead of the femoral artery. Uptake in the United States was slow, so Dr. Sunil Rao started a social media campaign with the hashtag #radialfirst, which resulted in more than 60,000 tweets by more than 7000 users, generating more than 100 million impressions within only 2 years [50]. Another popular hashtag is #dontdissthehis, promoting a certain form of pacemaker stimulation. Beer and colleagues have analyzed the uptake of this technique within the community and have seen it mirrored and likely amplified by the strong presence on twitter [51].

### … and cases

Sharing cases among colleagues and asking them for their input, be it of diagnostic nature (ECG, imaging) or regarding treatment recommendations, has been done for decades within the medical field. A famous quote by Sir William Osler reads, “Always note and record the unusual... When you have made and recorded the unusual or original observation … publish it” [52]. Social media represents a new paradigm of case discussions: a shear endless number of peers and experts to discuss with and the open-access nature of social media allow a “live” multispecialty discussion [53]. The possibility of sharing high-quality images and videos has allowed more comprehensive sharing and discussion of cases. Polls allow opinions on diagnostic and therapeutic approaches of the online colleagues to be collected. With knowledge accumulating more quickly than can be garnered by conventional means, social media provides a fast, enriched, multimedia-based way to learn and share medical knowledge. The COVID-19 pandemic provided the latest evidence of the usefulness of social media for the scientific medical field. Today, using social media can be seen as part of the professional skillset of a modern physician and scientist [54].

### Reaching out to patients

Ultimately, social media has the potential to be used to inform patients and to battle disinformation. Days or weeks dedicated to certain diseases that are heavily promoted on social media may have the potential to reach out and inform (undiagnosed) patients. On the international familial hypercholesterolemia (FH) awareness day, dedicated to a chronically underdiagnosed but life-threatening disease, a significant increase in FH-related twitter metrics was observed [55]. Finally, such outreach programs can be used for patient recruitment to clinical trials [56] outside of the classical hospital-centered clinical research atmosphere in so-called remote clinical trials fostered by the COVID-19 pandemic [57].

### Scientific opportunities

The fast and widespread uptake of wearables and mobile health applications by the public in combination with electronic health record data has generated immense data pools and unprecedented opportunities for research [58]. In addition, nationwide registries of diseases and other electronic health records together with available pharma claims data allow outcome research combined with detailed information regarding drug prescription and uptake without the immense costs of building and following up huge databases for clinical trials. Challenges, however, include handling, analyzing, interpreting, and securing such immense data collections. When done correctly, such data-driven analyses could potentially revolutionize the way we practice medicine and may introduce true personalized medicine.

COVID-19 has in some way revolutionized the way clinical trials may be conducted. In the classical clinic-based, randomized controlled trial approach, patients are recruited within a clinic and once enrolled, data and endpoint collection are done at the clinic with a large amount of additional trial visits necessary. While this certainly remains the gold standard, such trials are inflexible, cost a lot of money, and are lengthy, all aspects that render them unfeasible in a surging pandemic. Pragmatic, adaptive, and even remote trials were designed and rolled out at immense speed at the upcoming of the pandemic [59, 60]. About 1 month after the World Health Organization declared the COVID-19 outbreak a pandemic, first patients were remotely enrolled in a trial evaluating two pharmaceutical agents with potential beneficial effects [61]. Enrollment, randomization, medication dispersion, symptoms, COVID tests, and even ECGs were recorded remotely. Similar trials, including some within the healthcare workforce, followed rapidly [62–64].

## Health 4.0

The disruption of traditional healthcare systems caused by the COVID-19 pandemic has dramatically fueled innovations targeted at improving the quality and efficiency of care outside of the traditional office-based healthcare system [65]. Health 4.0 is a novel concept loosely based on the concept of Industry 4.0 or the Fourth Industrial Revolution, which describes the rapid change of technologies, industries, and societal patterns, blurring the lines between the physical, digital, and biological worlds. The idea of Health 4.0 builds on and leverages technologies such as artificial intelligence, gene editing, advanced robotics, quantum computing, an increasing interconnectivity between machines enabling smart automation, the emergence of the internet of things, ultrafast wireless internet, and augmented reality to provide a better, more efficient, and cost-effective healthcare for all [66]. It may integrate the internet of health things [67], medical cyber-physical systems [68], health cloud, and health fog [69], and combine big data analytics with machine learning, smart algorithms, and blockchain [70]. Six design principles have been described [70]: interoperability, virtualization, decentralization, real-time capabilities, service orientation, and modularity. Ultimately, Health 4.0 may disrupt the current healthcare business model and enhance interaction and improve flexibility, cost-effectiveness, and reliability, and ultimately translate to better healthcare and satisfied patients. Health 4.0 applications may target the patient, healthcare professionals, and healthcare systems. Building such applications is complex and requires reliable data collection and transfer as well as privacy and security operations. Ethical, legal, technical, and security issues need to be addressed globally and on a national basis to ensure the safe rollout of Health 4.0 application from which all patients should ultimately benefit. The COVID-19 pandemic provided a first opportunity to “beta-test” a few potential future applications and laid out potential applications for ongoing or potential future pandemics. These include the field of assisted diagnostics, augmented environments, disease predictions, and medical robotics that can be used in the prevention of contagion, improved diagnostics, digital teaching, and remote healthcare services [65].

Here, we can only provide a concise overview of some of the technologies currently being developed; for further reading, we recommend the references outlined in this section [70, 71].

## Conclusion and outlook

Digital transformations are changing all aspects of the life we know, including health and healthcare. They have the potential for both enormous, large-scale global benefits for all but also for disruptions in many areas. Designing proper governance of digital technologies should therefore be guided by public interests and not private profits, ensuring digital rights and trust in digital health and regulation of private partners [72]. A recently published thought piece by *The Lancet* and *Financial Times *commission on “governing health futures 2030: growing up in a digital world” has designated digital transformations as a key determinant of health [72]. With evidence gaps at the interface of digital technologies and health persisting, the commission argues for a precautionary, mission-oriented and value-based approach to the governance of digital transformations to succeed in improving health for all. Children and young people that grow up in a digital world and thus face the highest exposure to digital health need to be put at the center of attention. The collection and use of health data should be based on the concept of data solidarity protecting the individual’s rights while ensuring utilization of such data to serve the public good. Governments and other decision-makers are urged to create roadmaps and priorities as well as a general framework for digital transformation of healthcare. The immense potential of digital technologies in healthcare and associated research provides the opportunity for equitable and evidence-based care for all and may allow healthcare personnel to focus on their main task—the interaction with the patient.
